# Pyrrole-Pyridine and Pyrrole-Naphthyridine Hosts for Anion Recognition

**DOI:** 10.3390/molecules20069862

**Published:** 2015-05-27

**Authors:** M. Angeles García, M. Angeles Farrán, Dolores Santa María, Rosa M. Claramunt, M. Carmen Torralba, M. Rosario Torres, Carlos Jaime, José Elguero

**Affiliations:** 1 Departamento de Química Orgánica y Bio-Orgánica, Facultad de Ciencias, UNED, Paseo Senda del Rey 9, 28040-Madrid, Spain; E-Mails: afarran@ccia.uned.es (M.A.F.); dsanta@ccia.uned.es (D.S.M.); 2 Departamento de Química Inorgánica I and CAI de Difracción de Rayos-X, Facultad de Ciencias Químicas, UCM, 28040-Madrid, Spain; E-Mail: mtorres@quim.ucm.es; 3 Department de Química, Universitat Autònoma de Barcelona, 08193-Cerdanyola del Vallès, Spain; E-Mail: Carlos.Jaime@uab.cat; 4 Instituto de Química Médica, Centro de Química Orgánica Manuel Lora-Tamayo, CSIC, Juan de la Cierva 3, 28006-Madrid, Spain; E-Mail: iqmbe17@iqm.csic.es

**Keywords:** anion binding, NMR titrations, association constants, B3LYP/6-31G(d,p) calculations, X-ray structures

## Abstract

The association constants of the complexes formed by two hosts containing pyrrole, amide and azine (pyridine and 1,8-naphthyridine) groups and six guests, all monoanions (Cl^−^, CH_3_CO_2_^−^, NO_3_^−^, H_2_PO_4_^−^, BF_4_^−^, PF_6_^−^), have been determined using NMR titrations. The X-ray crystal structure of the host *N*^2^,*N*^5^-bis(6-methylpyridin-2-yl)-3,4-diphenyl-1*H*-pyrrole-2,5-dicarboxamide (**1**) has been solved (*P*2_1_/*c* monoclinic space group). B3LYP/6-31G(d,p) and calculations were carried out in an attempt to rationalize the trends observed in the experimental association constants.

## 1. Introduction

The design of receptors for selective recognition of anions, by means of weak interactions such as hydrogen bonding, is still an active area of research within supramolecular chemistry, in view of their large number of applications [1,2,3,4,5,6,7,8]. Although most biological reactions occur in an aqueous environment, it is also true that anion recognition in ion channels or enzyme active sites takes place in hydrophobic environments and simple model systems could be helpful to understand their mode of action [9].

**Figure 1:**
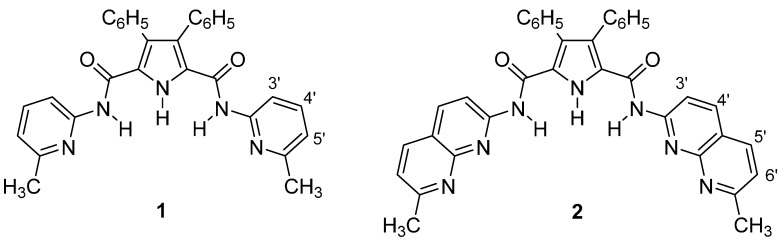
Structures of the hosts **1** and **2**.

As our group has been involved for several years in the synthesis and molecular recognition studies of a family of receptors for urea derivatives [10], we decided to take a step forward and study their binding properties towards a series of anions (Cl^−^, CH_3_CO_2_^−^, NO_3_^−^, H_2_PO_4_^−^, BF_4_^−^, PF_6_^−^), in low polarity solvents. For this purpose, two hosts **1** and **2** were selected, that combine a central pyrrole ring with a NH group and two side arms, 2-methylpyridine and 7-methyl-1,8-naphthyridine, bonded through amide groups, as shown in Figure 1. We have reported their synthesis and binding to ureas in [10]. Receptors with similar structures were previously investigated by Gale (different kinds of anions in DMSO-water mixtures) [11,12] and by Zieliński and Jurczak (amides and thioamides) [13]. 

The above-mentioned hosts, **1** and **2**, gave promising results for urea derivatives [10] and therefore they were selected to test complexation with anions. Although *a priori* it might seem that the presence of the 2-methylpyridine or the 7-methyl-1,8-naphthyridine rings would hamper anion binding (other authors use alkyl or aryl rings [11,12,13]), we imagined that such substituents would preferentially recognize the dihydrogen phosphate anion since it has acid O–H groups that could form hydrogen bonds with the pyridinic N atoms, thus resulting in some selectivity. 

## 2. Results and Discussion

We report here the complexation studies of *N*^2^,*N*^5^-bis(6-methylpyridin-2-yl)-3,4-diphenyl-1*H*-pyrrole-2,5-dicarboxamide (**1**) and *N*^2^,*N*^5^-bis(7-methyl-1,8-naphthyridin-2-yl)-3,4-diphenyl-1*H*-pyrrole-2,5-dicarboxamide (**2**) with six monoanions of five different shapes: (i) spherical like Cl^−^; (ii, iii) trigonal planar or V-shaped like CH_3_CO_2_^−^ and NO_3_^−^; (iv) tetrahedral like H_2_PO_4_^−^ and BF_4_^−^; (v) octahedral like PF_6_^−^, all in the form of tetrabutylammonium salts.

The preparation of hosts **1** and **2**, achieved by condensation of the 3,4-diphenyl-1*H*-pyrrole-2,5-dicarbonyl dichloride with 2-amino-6-methylpyridine and 2-amino-7-methyl-1,8-naphthyridine, as well as their complete NMR characterization is reported elsewhere [10]. The most significant proton signals used in the NMR titrations are the pyrrole NH singlet at 10.44 ppm, the amide NH singlet at 7.97 ppm and the H3' doublet at 7.99 ppm in host **1** and at 10.54 ppm, 8.37 ppm and 8.50 ppm in host **2**.

**Figure 2:**
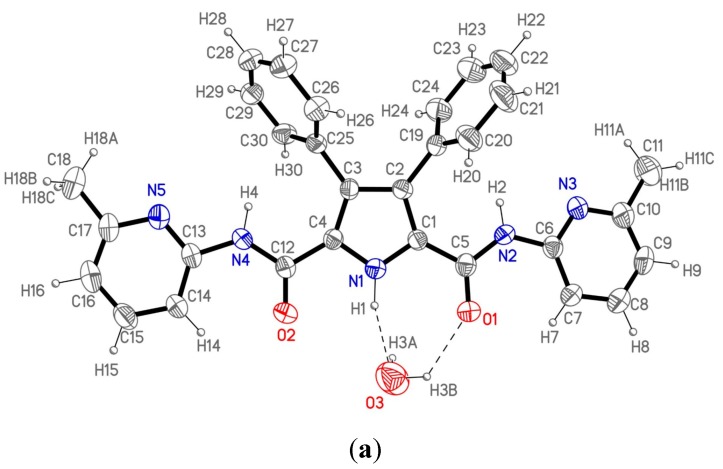

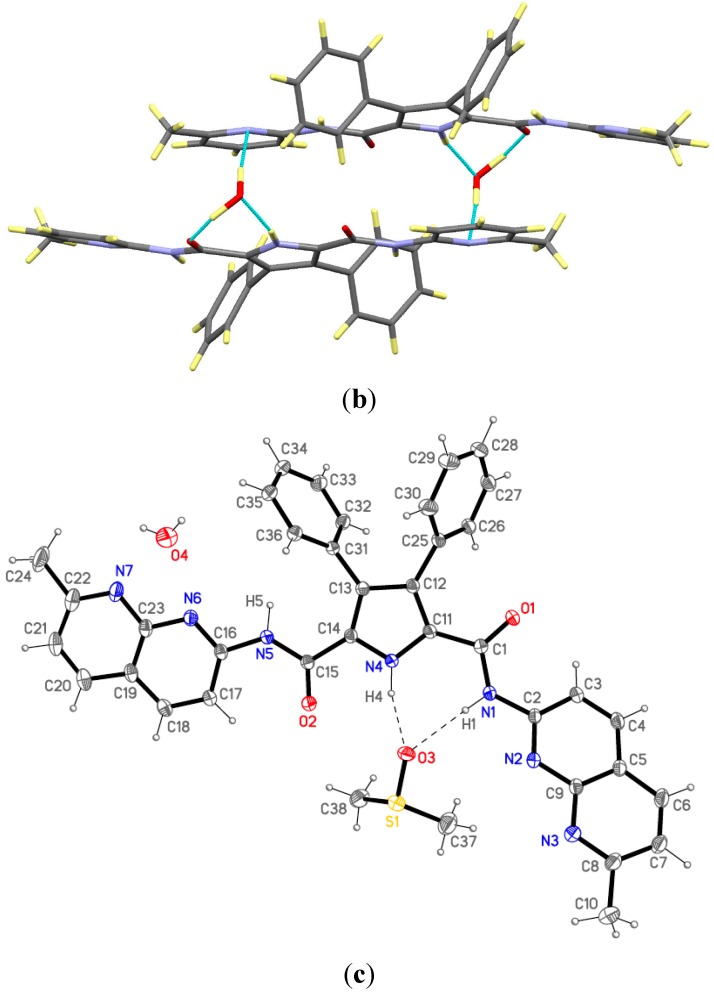
(**a**) ORTEP plot (40% ellipsoid probability) of 1 showing the labeling of the asymmetric unit; (**b**) View of the 1 dimer (1_2_); (**c**) The structure of 2 with a water and a DMSO molecule from reference [10].

Crystals of host **1** suitable for analysis by single crystal X-ray diffraction were obtained by recrystallization either from chloroform-hexane or from ethanol. It crystallizes in the monoclinic *P*2_1_*/c* space group. Figure 2a shows the labeling of the asymmetric unit and H-bonding data are collected in Table 1.

**Table 1:** Hydrogen bonds (Å and °) for compound **1**.

D-H···A	Symmetry Operation	d(D-H)	d(H···A)	d(D···A)	<(DHA)
N1-H1···O3		1	2.18	3.151(5)	162.5
O3-H3B···O1		1.07	2.09	2.855(4)	126.2
O3-H3A···N5(#1)	(#1) –x + 1, –y, –z	1.05	2.07	3.097(5)	165.6

The asymmetric unit consists of a single molecule of **1** and one crystallization water molecule. The molecule is not planar due to the twisted 3,4-disubstituted phenyl rings with respect to the pyrrole moiety; the dihedral angle between the latter and each of the two mentioned rings is 60.2(3)° and 66.9(3)°, respectively. The rest of the molecule could be considered nearly planar as the atoms in the pyrrole ring, the amido groups and the pyridine rings show an extended electronic conjugation in accordance with the bond distances found in this part of the molecule.

These molecules are not isolated as each one connects with the centrosymmetric one through two different water molecules appropriately situated to form hydrogen bonds (Figure 2b) giving rise to a dimeric unit. Therefore, each dimeric entity consists of two host molecules linked by two bridging water molecules. Each water molecule forms three different hydrogen bonds with the two hosts in the dimeric unit. So, every water molecule interacts with the first one through a double hydrogen bond O3···N1–H1 and O3–H3B···O1 (the pyrrolic and the amido group respectively), and the third hydrogen bond is formed with the second molecule through O3-H3A and the N5’ atom, completing the dimeric moiety. These dimers are isolated, as no significant additional interactions between them have been found in the crystal structure.

When comparing the molecular structure of **1** (Figure 2a) with that of host **2** already described by us in reference 10 (Figure 2c) immediately the role of DMSO in changing the conformation of one of the lateral heterocycles (in this case, a 1,8-naphthyridine) is apparent. In its absence, one must expect a conformation similar to that of **1**.

The binding properties have been determined by ^1^H-NMR titrations in deuterochloroform following the complexation effects on the chemical shifts (CIS) of the amide NH (and the pyrrole NH for BF_4_^−^) and H3*'* signals of the hosts **1** and **2**, by addition of the anions as their tetrabutylammonium salts, and the association constants *K_a_* are reported in Table 2. It is important to note that the acidic protons bonded to the N atoms disappear when the anion is added even, in some cases, at the first 10 μL addition of the solution containing the anion. The pyrrolic N–H is the first to disappear (it is only observed for the **1/**BF_4_^−^ combination), then both amidic N–Hs that could be used for the titration only with the acetate anion. This is an indication that, in some cases, there are equilibria involving host anions and neutral guests. 

**Table 2:** Experimental determination of the association constants *K_a_* (M^−1^).

Host	Guest	H3*'*	Amide NH	Mean
**1**	BF_4_^−^	702 ± 22	595 ^a^ ± 123	648.5
**1**	CH_3_CO_2_^−^	37 ± 8	24 ± 5	30.5
**1**	NO_3_^−^	0	---	
**1**	H_2_PO_4_^−^	10005 ± 65	---	
**1**	PF_6_^−^	0	---	
**1**	Cl^−^	0	---	
**2**	BF_4_^−^	0	---	
**2**	CH_3_CO_2_^−^	312 ± 48	294 ± 30	303
**2**	NO_3_^−^	0	---	
**2**	H_2_PO_4_^−^	440 ± 47	---	
**2**	PF_6_^−^	0	---	
**2**	Cl^−^	3748 ± 106	---	

^a^ Using the pyrrole N–H the value is 560 ± 153.

As we hypothesized in the introduction, the presence of the 2-methylpyridyl substituent makes receptor **1** highly selective for H_2_PO_4_^−^ since it does not recognize NO_3_^−^, PF_6_^−^ and Cl^−^ and only binds moderately to BF_4_^−^ and very weakly with CH_3_CO_2_^−^. However, the behavior of receptor **2** is different and this needs an explanation that we will provide later on.

The experimental values of *K_a_* have been transformed into Δ*G* values (Δ*G* = −RT Ln *K_a_*, R = 8.314 J·K^−1^·mol^−1^, T = 298.15 K). Anions for what *K_a_* values could be determined, which excludes the nitrate and hexafluorophosphate anions, are reported in Table 3.

The lowest *K_a_* that has been determined corresponds to **1**·CH_3_CO_2_^−^ (30.5 M^−1^); in the cases of **1**·Cl^−^ and **2**·BF_4_^−^*K_a_* is not equal to zero but a lower value, that we have assumed *K_a_* = 10 corresponding to Δ*G =* −5.7; but this value is to be considered with caution.

We have calculated the total Gibbs free energies (*G* in Hartree) of the three species, the host H, the guest G and the complex C, and from these values we have calculated Δ*G* (C-H-G): *G*_Complex_ − *G*_Host_ − *G*_Guest_ (Table 3), for the six most interesting cases.

**Table 3:** The experimental and calculated host-guest interaction energies (Δ*G*) (underlined, assumed values). All Δ values in kJ·mol^−1^.

Host	Guest	Exp. *K_a_*	Exp. Δ*G*	Calc. Δ*G*
1	BF_4_^−^	648.5	−16.1	−57.2
1	CH_3_CO_2_^−^	30.5	−8.5	−131.8
1	H_2_PO_4_^−^	10005	−23.0	−104.6
2	BF_4_^−^	10	−5.7	−69.3
2	CH_3_CO_2_^−^	303	−14.3	−140.2
2	H_2_PO_4_^−^	440	−15.2	−75.9

The calculations were carried out at the B3LYP/6-31G(d,p) level (see Section 3.3. Computational Details). The host, when isolated, was supposed having a *C*_2_ symmetry with a geometry preorganized for the complexation. In some cases, to favor the complexation with some guests, like the acetate anion, *C_s_* symmetry of the host was considered, however at the end of the optimization process, the *C*_2_ geometry was again obtained.

**Figure 3:**
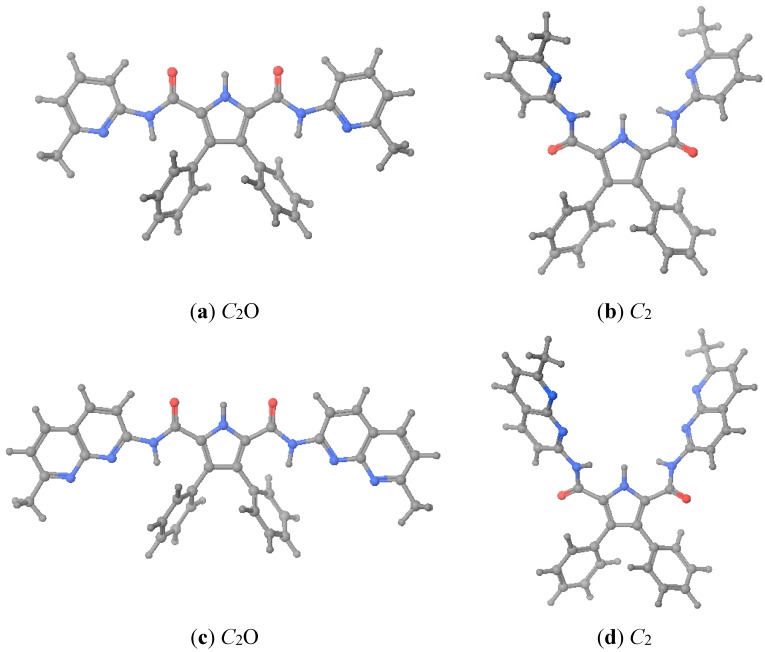
Optimized geometries of hosts **1** and **2**. (**a**) **1**·X-ray; (**b**) **1**·preorganized; (**c**) **2**·X-ray; (**d**) **2**·preorganized. *C*_2_O is the geometry where the C=O of the urea groups pointed towards the pyrrole NH.

As described in the foregoing section, hosts **1** and **2** crystallized with geometries different from that was assumed as preorganized. When we calculated the energies corresponding to the X-ray geometries (close to *C*_2_O, see Figure 3), we found that, after optimization, they were 54–58 kJ·mol^−1^ more stable (see Table 4). Therefore, when we calculated the complexation energies we have used the energies corresponding to the more stable X-ray geometries. Note that the *G*_rel_ values are lower than the *E*_rel_ ones.

**Table 4:** Energies (*E*) and free energies (*G*) in hartrees as well as corresponding energy differences in kJ·mol^−1^ of hosts **1** and **2** using as starting points the X-ray and preorganized geometries.

Host	Structure	*E* (Hartrees)	*E*_rel_ (kJ·mol^−1^)	*G* (Hartrees)	*G*_rel_ (kJ mol^−1^)
**1**	X-ray	−1582.58265	0.0	−1582.15847	0.0
****	preorganized	−1582.56048	58.2	−1582.13729	55.6
**2**	X-ray	−1921.95091	0.0	−1921.45682	0.0
****	preorganized	−1921.93051	53.6	−1921.44290	36.5

Due to the difficulty of obtaining a geometry of the complex with the lowest possible energy, consequence of the high number of degrees of freedom of these systems, the different complexes were built up maximizing the number of stabilizing interactions between host and guest, especially, the maximum number of hydrogen bonds. The calculated free energy results are shown in Table 5.

**Table 5:** Results of the B3LYP/6-31G(d,p) calculations. The column reporting the symmetry of the complex indicates the geometry of the closest one, e.g., *C*_2_ means that the geometry is close to *C*_2_.

Complex	*G* (Host) (Hartrees)	*G* (Guest) (Hartrees)	*G* (Complex) (Hartrees)	Symmetry	Δ*G* (C-H-G) (Hartrees)	Δ*G* (kJ·mol^−1^)
**1**·BF_4_^−^	−1582.15847	−424.51203	−2006.69234	*C*_2_	−0.0218	−57.2
**1**·CH_3_CO_2_^−^	−1582.15847	−228.48139	−1810.69007	*C*_2_	−0.0502	−131.8
**1**·H_2_PO_4_^−^	−1582.15847	−643.59035	−2225.78867	*C_sh_*	−0.0398	−104.6
**2**·BF_4_^−^	−1921.45682	−424.51203	−2345.99517	*C*_2_	−0.0264	−69.3
**2**·CH_3_CO_2_^−^	−1921.45682	−228.48139	−2149.99162	*C*_2_	−0.0534	−140.2
**2**·H_2_PO_4_^−^	−1921.45682	−643.59035	−2565.07605	*C_s_O*	−0.0289	−75.9

**Figure 4:**
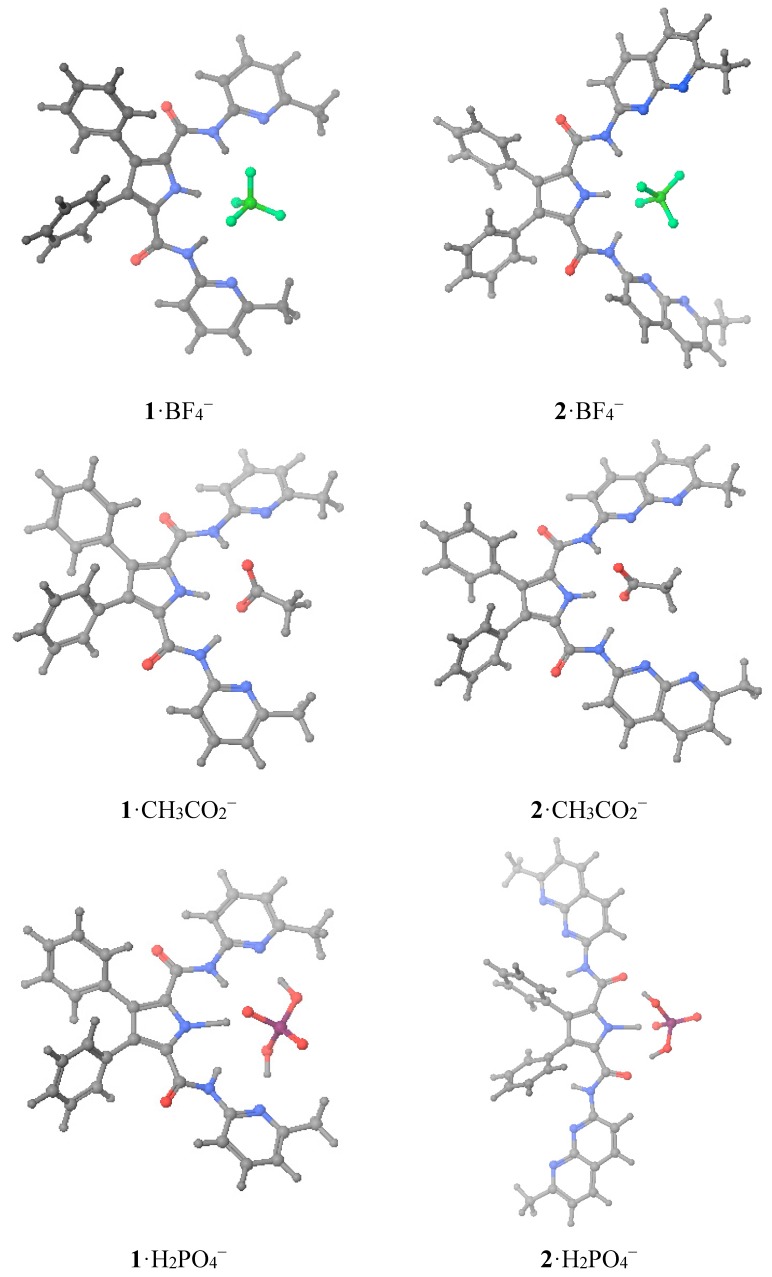
Optimized geometries of the six studied complexes.

That is, we have theoretically calculated both hosts **1** and **2** (Table 4) plus the complexes they formed with three monoanions, BF_4_^−^, CH_3_CO_2_^−^ and H_2_PO_4_^−^ (Table 5) whose structures are represented in Figure 4. Interestingly, a total of three energetic minima were obtained for the complex **1**· H_2_PO_4_^−^, two with *C_2_* geometry and the other with a *C_s_O* geometry (see Figure 3). Both Figure 4 and Table 6 depict the calculated as the most stables geometries.

A very interesting result of Figure 4 and Table 6 concerns the structures **1**·H_2_PO_4_^−^ and **2**·H_2_PO_4_^−^. For the pyridinic derivative (host **1**), the phosphate not only binds to the pyrrolic N–H by the negatively charged O atom, but also forms two O–H···N HBs with the basic picoline N atoms. This results in a very strong interaction (Δ*G*_exp_ = −23.0 kJ·mol^−1^) with the proton of the pyrrole partly transferred to the anion (see Table 6). The situation of the **2**·H_2_PO_4_^−^ is very different: here the methylnaphthyridine does not play any role and the interaction is sustained by three HBs: one N–H···O^(−)^ and two O–H···O, which is much less efficient (Δ*G*_exp_ = −15.2 kJ·mol^−1^) , although the pyrrolic N–H is similarly elongated. The different behavior of the two hosts is probably related to the fact that pyridine is a much stronger base than 1,8-naphthyridine, 5.20 *vs.* 3.39 p*K*_a_ units [14]. In the free host, the N–H bond lengths of the pyrrole and the amides are 1.009 and 1.012 Å for the C_2_O and 1.012 and 1.014 Å for the C_2_, respectively. The formation of the complexes results in a small elongation of the pyrrolic and amide NHs, the usual effect of the hydrogen bonds. In the complex **1**^.^BF_4_^−^ the pyrrole N–H length is 1.026 Å, the shortest of all the complexes (the same happens for the amide N–H bond lengths); this could account for the observation of this proton during the titrations (Table 2).

The only exception concerns the dihydrogen phosphate anion where a considerable lengthening is observed, indicative of a proton partly transferred from the pyrrole to the guest: dN(pyrrole)^−^···O_4_PH_3_.

**Table 6:** Calculated geometries of the hydrogen bonds. 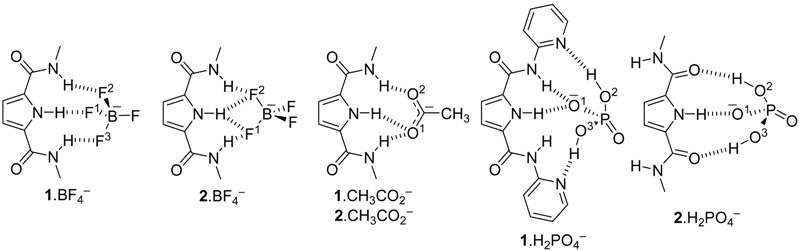

Host	Guest	Distances (Å)	Angles (°)
**1**	BF_4_^−^	dN(pyrrole)–H = 1.026	(pyrrole)N–H···F^1^ = 158.4
		dN(amide)–H = 1.026	(amide)N–H···F^2^ = 167.5
		dF^1^···H–N(pyrrole) = 1.752	(amide)N–H···F^3^ = 160.4
		dF^2^···H–N(amide) = 1.774	
		dF^3^···H–N(amide) = 1.823	
**2**	BF_4_^−^	dN(pyrrole)–H = 1.026	(pyrrole)N–H···F^1^ = 124.7
		dN(amide)–H = 1.024	(pyrrole)N–H···F^2^ = 164.2
		dF^1^···H–N(pyrrole) = 2.214	(amide)N–H···F^1^ = 158.5
		dF^2^···H–N(pyrrole) = 1.703	(amide)N–H···F^2^ = 163.4
		dF^1^···H–N(amide) = 1.812	
		dF^2^···H–N(amide) = 1.864	
**1**	CH_3_CO_2_^−^	dN(pyrrole)–H = 1.071	(pyrrole)N–H···O^1^ =167.1
		dN(amide)–H = 1.041	(amide)N–H···O^1^ =171.8
		dO^1^···H–N(pyrrole) = 1.562	(amide)N–H···O^2^ =175.0
		dO^1^···H–N(amide) = 1.785	
		dO^2^···H–N(amide) = 1.743	
**2**	CH_3_CO_2_^−^	dN(pyrrole)–H = 1.086	(pyrrole)N–H···O^1^ =168.6
		dN(amide)–H = 1.045	(amide)N–H···O^1^ =172.6
		dO^1^···H–N(pyrrole) = 1.511	(amide)N–H···O^2^ =173.5
		dO^1^···H–N(amide) = 1.747	
		dO^2^···H–N(amide) = 1.694	
**1**	H_2_PO_4_^−^	dN(pyrrole)–H = 1.727	(pyrrole)N–H···O^1^ = 161.1
		dN(amide)–H = 1.016	(amide)N–H···O^1^ = 170.1
		dO^1^···H–N(pyrrole) = 1.014	O^2^–H···N(pyridine) = 169.8
		dO^1^···H–N(amide) = 1.921	O^3^–H···N(pyridine) = 166.6
		dO^2^–H···N(pyridine) = 1.753	
		dO^3^–H···N(pyridine) = 1.694	
**2**	H_2_PO_4_^−^	dN(pyrrole)–H = 1.723	(pyrrole)N–H···O^1^ = 173.5
		dO^1^···H–N(pyrrole) = 1.006	O^2^–H···O=C(amide) =169.1
		dO^2^–H···O=C(amide) = 1.844	O^3^–H···O=C(amide) =176.7
		dO^2^–H···O=C(amide) = 1.807	

The calculated Δ*G* values of Table 3 and Table 5 show that there is a very good relationship between the values of both hosts for the same guests in the case of BF_4_^−^ and CH_3_CO_2_^−^ anions (**2**/**1** ratio ≈ 1.1) but not for the H_2_PO_4_^−^ anion (**2**, −75.9; **1**, −104.6 kJ·mol^−1^, ratio ≈ 0.7). This is a very significant result. In absolute values, calculated ∆*G* varies in the order CH_3_CO_2_^−^ >> H_2_PO_4_^−^ > BF_4_^−^. This is the order found in some experimental studies, for instance using thiosemicarbazide neutral sensors [15]. The tetrafluoroborate anion is often used for cationic host recognition of anions because it has no affinity for the receptors [16,17].

The experimental ∆*G* values ordering depends on the host: **1**, H_2_PO_4_^−^ > BF_4_^−^ >> CH_3_CO_2_; **2**, H_2_PO_4_^−^ > CH_3_CO_2_^−^ >> BF_4_^−^. Thus, they are different and none follow the calculated values. Why so? The experimental value of **1**·BF_4_^−^ (−16.1 kJ·mol^−1^) is probably overestimated and that of **2**·BF_4_^−^(−5.7 kJ·mol^−1^) underestimated, thus the correct order for both hosts would be H_2_PO_4_^−^ > CH_3_CO_2_^−^ >> BF_4_^−^. Thus compared with the calculated order, there is an inversion between acetate and dihydrogenphosphate. There are two possible explanations, one experimental and the other theoretical. Although we have used a deuterochloroform of the best available quality (stored over silver wire, see Experimental, to prevent the formation of DCl), traces of water cannot be avoided, and if present they could modify the *K_a_* values in a non-predictive way (specific solvation). The H_2_PO_4_^−^ anion, according to the calculations, is able to deprotonate the receptor in the gas phase, leading to an anion host plus phosphoric acid (this a well-known phenomenon [4,15]), at least partially, and this is an acid-base equilibrium difficult to assess in solution. These explanations, alone or in combination, are the probable cause of the discrepancies between experiments and calculations. What it is the most important theoretical result is that both receptors are very similar when hosting CH_3_CO_2_^−^ and BF_4_^−^ but very different towards H_2_PO_4_^−^ explaining why **1** is highly selective for H_2_PO_4_^−^, contrarily to **2**.

## 3. Experimental Section

### 3.1. Materials

The tetrabutylammonium salts of the six monoanions are commercially available: Bu_4_N^+^Cl^−^ (99%), Bu_4_N^+^CH_3_CO_2_^−^ (97%), Bu_4_N^+^NO_3_^−^ (97%), Bu_4_N^+^H_2_PO_4_^−^ (97%), Bu_4_N^+^BF_4_^−^ (99%), Bu_4_N^+^PF_6_^−^ (98%). Hosts and guests were dried under vacuum at 60 °C for 24 h. 

### 3.2. NMR Titrations 

**Figure 5:**
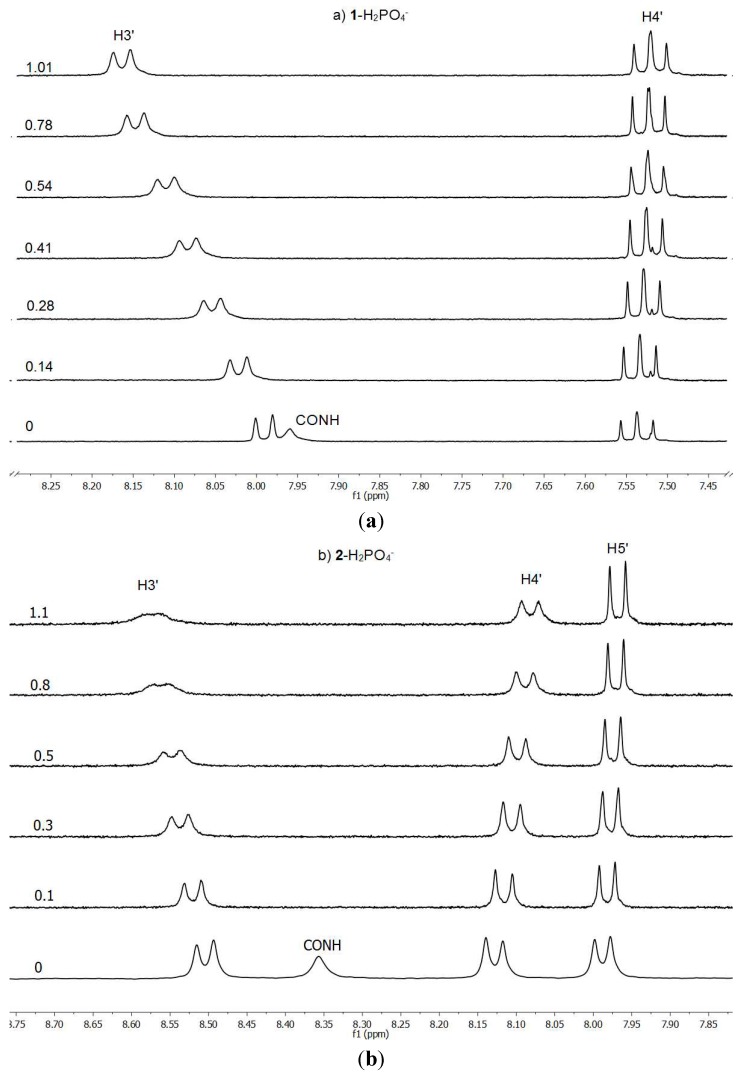
Evolution of the ^1^H-NMR spectra in CDCl_3_: (**a**) of **1** upon addition of H_2_PO_4_^−^; (**b**) of **2** upon addition of H_2_PO_4_^−^, from 0 to 1.1 equiv.

^1^H-NMR spectra were recorded on a DRX 400 (9.4 T, 400.13 MHz) spectrometer (Bruker Española S.A., Madrid, Spain) at 300 K. The [Host] values in the range of 1.02 to 1.85 × 10^−3^ correspond to a weighted quantity of host in 2 mL of CDCl_3_ (S33657, deuterium content >99.8%, water content <0.01%, containing silver wire as stabilizer, Merck S.L., Mollet del Vallés-Barcelona, Spain). A given quantity of the guest (about 2 × 10^−2^ M) was weighed in a 1 mL volumetric flask and host solution was added to reach the graduation mark; in this way we know that the host concentration remains constant. Host and guest were weighted in a AE260-Delta Range scale (error ± 0.00005 g, Mettler Toledo, L*'* Hospitalet de Llobregat-Barcelona, Spain) and eVol^®^ XR hand-held automated analytical syringes (500 µL, 50 µL) from SGE Analytical Science (Trajan Scientific Europe Ltd, Crownhill, Milton Keynes, United Kingdom) previously calibrated for CDCl_3_, were used to perform additions. ^1^H-NMR titrations were used to quantify *K_a_* values (see Figure 5 for two representative plots).

These titrations were carried out monitoring the Chemical Induced Shift (CIS) in one or more host protons (H3*'*, CONH and pyrrole NH for BF_4_^−^) as the concentration of the formed complex varies upon addition of one of the components. Each experiment was repeated thrice getting similar values of *K_a_*. The results are reported in Table 2. Among the large number of ways to fit data from a titration [18], non-linear curve fitting is generally accepted as the method with the lowest error in the determination of *K_a_* values, in comparison to others that employ approximations to reach a linear relationship between some physical property and *K*_a_ [19]. Binding curves were fit using the Wineqnmr program [20]. The quality of fit was estimated using the merit-function shown in Equation (1) where w_i_ is the weight attributed to observation i (normally data points were assigned equal weights) [21]:

*R* = 100 × {[Σw_i_(*d*_obs_ − *d*_calc_)^2^]/Σw_i_(*d*_obs_)^2^}^1/2^ (1)

The basic equation for this kind of titrations is represented in Equation (2), showing the relationship between chemical shifts (δ), concentrations of host H, guest G and complex C, and the association constant *K*_a_ = [HG]/([H][G]). This equation is valid only for 1:1 stoichiometry:

∆*d* = δ_HG_ ([HG]/[H]_o_) (2)

**Figure 6:**
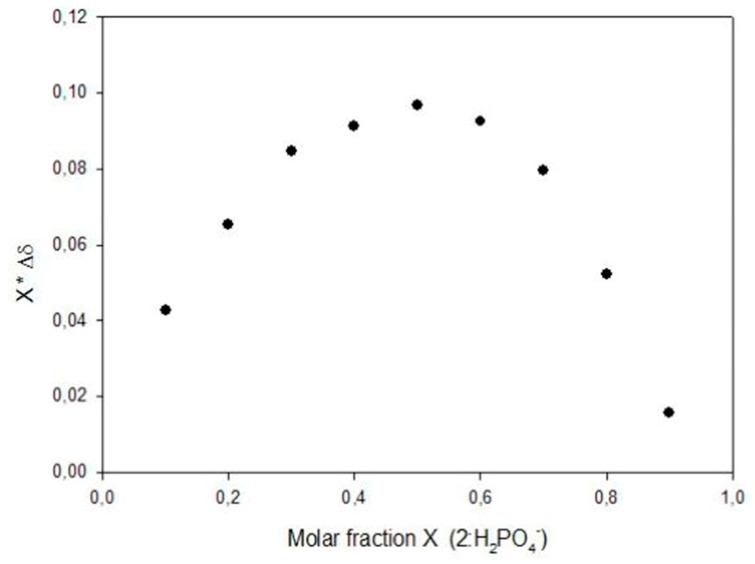
Job plot titration for **2**: H_2_PO_4_^−^ (3 mM).

The stoichiometry of the host-guest complexes was determined by the continuous variation method [22,23,24]. Both stock solutions of the hosts (3 mM) and guests (3 mM) were prepared in a n/m (% *v*/*v*) mixture of CDCl_3_. To obtain the desired host-guest ratios, which varied from 0 to 1 (molar fraction of **1** and **2**), appropriate volumes of stock solutions were mixed. In a final volume equal to 500 µL, the sum of host and guest concentrations is constant (3 mM). From the value of the maximum, which can be obtained by means of equation X = m/(m + n), the stoichiometry of the complex is determined: in all cases it was 1:1. A representative curve is shown in Figure 6.

### 3.3. Computational Details

All calculations were carried out using the Maestro programs package [25]. The structures of the hosts were built up using the graphic tools available in Maestro. Initially the geometries were optimized by molecular mechanics using the MM3* [26,27,28] force field within the MacroModel program [29,30], included in Maestro. Afterwards, the energy was minimized and the geometry optimized using a DFT method, namely, the B3LYP/6-31G(d,p) [31,32,33,34,35]. All the quantum mechanics calculations were carried out with the Jaguar program [36], also within the Maestro package. The guests were treated in the same way. The complexes were built up joining the optimized structures of the host and the guest. The tools in Maestro allow orienting the guest inside the host until a reasonable geometry is obtained, a geometry that was subjected to energy minimization and geometry optimization at the B3LYP/6-31G(d,p) level [27,28,29,30,31,32,33,34] using Jaguar [36]. The results are reported in Table 5, where Δ*G* (C-H-G): *G*_Complex_ − *G*_Host_ − *G*_Guest_. In no case, symmetry restrictions were imposed, and all geometries were computed to be true minima (positive vibrational frequencies).

### 3.4. X-ray Data Collection and Structure Refinement

Data collection was carried out at room temperature on a Bruker Smart CCD diffractometer using graphite-monochromated Mo-Kα radiation (λ = 0.71073 Å) operating at 50 kV and 25 mA. The data were collected over a hemisphere of the reciprocal space by combination of three exposure sets. Each exposure of 20 s covered 0.3 in ω. The cell parameters were determined and refined by a least-squares fit of all reflections. The first 100 frames were recollected at the end of the data collection to monitor crystal decay, and no appreciable decay was observed. A summary of the fundamental crystal and refinement data is given in Table 7.

**Table 7:** Crystal data and structure refinement for host **1**.

CCDC	1045562
Empirical formula	[C_30_H_27_N_5_O_3_]
Formula weight	505.57
Crystal system	Monoclinic
Space group	*P*2_1_/c
Space group number	14
*a*/Å	8.319(1)
*b*/Å	16.584(3)
*c*/Å	19.617(3)
α (°)	90.0
β (°)	98.994(3)
γ (°)	90.0
*V*/Å^3^	2673.2(7)
*Z*	4
*F*(000)	1064
ρ_c_/g·cm^−3^	1.256
μ/mm^−1^	0.083
*Data collected*	(−9, −19, −23) to (9, 16, 23)
*θ range (°)*	1.62 to 25.01
*Reflections collected*	20093
*Independent reflections*	4675 (Rint = 0.0883)
*Completeness to maximum θ (%)*	99.4
*Data/restraints/parameters*	4675/0/343
*Observed reflections [I> 2σ(I)]*	1961
*R ^a^*	0.0616 (6.2%)
*RwF ^b^*	0.1790

*^a^* Σ[|*F_o_*| −|*F_c_*|]/Σ[|*F_o_*|; *^b^* {Σ[w(*F_o_*^2^−*F_c_*^2^)^2^] / Σ[w(*F_o_*^2^)^2^]}^1/2^.

The structure was solved by direct methods and refined by full-matrix least-square procedures on F^2^ (SHELXL-97)* [37]. All non-hydrogen atoms were refined anisotropically. The hydrogen atoms were included in their calculated positions and refined riding on the respective carbon atoms with the exception of hydrogen atoms H3A and H3B bonded to O3, H1 bonded to N1, H2 bonded to N2 and H4 bonded to N4 that were located in a Fourier synthesis and refined riding on the respective bonded atoms.

## 4. Conclusions

A series of association constants *K_a_* have been determined by ^1^H-NMR titration experiments in deuterochloroform. In all cases, the host-guest stoichiometry was 1:1. The measured ∆G values range from −8.5 to −23.0 kJ·mol^−1^.

The X-ray structure of one host, *N*^2^,*N*^5^-bis(6-methylpyridin-2-yl)-3,4-diphenyl-1*H*-pyrrole-2,5-dicarboxamide (**1**), was determined and the geometry used as starting point for the theoretical calculations. Calculations, at the B3LYP/6-31G(d,p) level, of the host-guest interaction free energies differ from the experimental association constants in what concerns acetate and dihydrogenphosphate monoanions.

We have found that receptor **1** is highly selective for H_2_PO_4_^−^ since it does not recognize NO_3_^−^, PF_6_^−^ and Cl^−^ and only binds moderately with BF_4_^−^ and very weakly with CH_3_CO_2_^−^. The calculated geometries and their associated energies account for this observation.

## Supporting Information

CCDC-1045562 contains the supplementary crystallographic data for this paper. These data can be obtained free of charge from The Cambridge Crystallographic Data Centre via www.ccdc.cam.ac.uk/data_request/cif.
